# Breeding for improved heat tolerance in dairy cattle: Methods, challenges, and progress*

**DOI:** 10.3168/jdsc.2024-0651

**Published:** 2024-12-16

**Authors:** Ignacy Misztal, Luiz F. Brito, Daniela Lourenco

**Affiliations:** 1 Animal and Dairy Science, University of Georgia, Athens, GA 30602; 2 Department of Animal Sciences, Purdue University, West Lafayette, IN 47907

## Abstract

Heat tolerance in dairy cattle has likely deteriorated over time due to unfavorable genetic correlations between milk production level and heat tolerance, with stronger deterioration in later parities. The dairy cattle industry has responded to the challenge of heat stress by implementing management modifications. Methodology exists to calculate genetic prediction of breeding values for high production under heat stress conditions, with high reliabilities of breeding values obtained when incorporating genomic information. However, cows that maintain production during the heat stress peak have an increased likelihood of death. One remedy would be selection for better fertility and survival under heat stress, but with a low volume of data and low heritabilities, corresponding reliabilities may be too low for an efficient selection. In environments where intensive management is too expensive, an ideal cow would maintain production in a favorable climate, would briefly reduce production during heat stress, and would restore production after the heat stress conditions are over. As there are many biological mechanisms involved in heat stress response, in addition to deriving heat tolerance indicators based on variability in performance traits under heat stress conditions, novel traits that capture physiological, behavioral, and anatomical traits related to heat stress response, less dependent on production level, could also contribute to breeding for improved heat tolerance.

## Highlights

• Current genetic selection acts against heat tolerance.
• Management for mitigating heat stress is continually improved.
• Required level of genetics for heat tolerance depends on management intensity.
• Improved heat tolerance can be obtained by stronger selection on fitness traits.
• There is a possibility of selection for resilient cows that drop production when needed.

## Graphical Abstract

**Figure:**
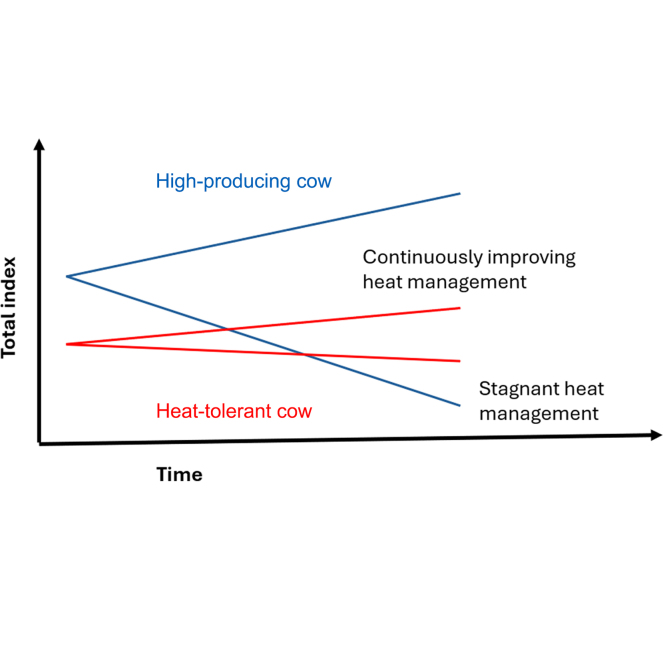
**Summary:** This article looks at possible methods to select heat-tolerant cows. Direct selection for better performance under heat stress is possible but would reduce yields. Indirect selection would include greater weights on fertility, health, and livability, with weights dependent on herd management. Selection for a more resilient cow is a possibility.

Heat stress conditions have caused large economic losses across species for many years (St-Pierre et al., 2003), in addition to animal welfare issues. High-producing dairy cows are more sensitive to heat stress due to the challenge of dissipating the greater metabolic heat production under heat stress conditions (Spiers, 2012). Under heat stress, production and fertility tend to drop, and cows are more vulnerable to diseases that can end in sudden death (West, 2003). For instance, intensive selection for milk production and conformation traits have led to unfavorable genetic responses on fertility and health traits (Brito et al., 2021). However, a recent meta-analysis of genetic parameters in worldwide Holstein cattle indicated large variability in the relationships between resilience indicators and productivity (Maskal et al., 2024). To mitigate heat stress, the dairy industry uses extensive heat mitigation strategies (Collier and Collier, 2012). Except in Australia (Nguyen et al., 2016), dairy cows are not directly selected for improved heat tolerance. Due to continued global warming, cows are expected to experience stronger and longer heat waves (e.g., https://www.c2es.org/content/heat-waves-and-climate-change/). Even areas with previously moderate climates are now experiencing heat stress (e.g., Canada; Campos et al., 2022). The wide adoption of genomic selection in the worldwide dairy cattle industry brings new capabilities for accelerated genetic improvement in traits that are more difficult or expensive to measure (Weller et al., 2017).

The need for breeding dairy cattle populations for improved heat tolerance while maintaining high productivity is evident, but the methods, indicator traits, and selection strategies are still being investigated. There are many indigenous breeds that are well adapted to hot environments; however, their milk production is lower than that of more specialized dairy breeds (e.g., Holstein). Crossbreeding between locally adapted and more specialized dairy breeds has also been used as a strategy for developing more productive and better-adapted dairy populations, such as the Brazilian Girolando breed, which is a cross between Holstein (*Bos taurus taurus*) and Gyr (*Bos taurus indicus*) (Negri et al., 2023). However, in this symposium article, we will focus on the methods developed, challenges, and progress achieved toward breeding for improved heat tolerance in high-producing dairy cattle breeds.

The main methods to study genetics of heat tolerance in livestock as well as their issues and practical experiences were reviewed by Misztal (2017). Most studies investigating the genetic background of heat tolerance have used existing phenotypes on production or fertility associated with temperature-humidity index (**THI**) values obtained from public weather stations. This association can be modeled using a broken line model where a cow's performance is constant until a THI threshold, and then is assumed to slope linearly; the rate of the slope is interpreted as heat tolerance breeding value (**HTBV**). Generally, the slope has an additive genetic variance and is antagonistic to production under no heat stress. As a result, daughters of more heat-tolerant bulls tend to produce less milk but are superior for fertility, survival, and udder depth (Bohmanova et al., 2008; Jensen et al., 2022). Interestingly, studies with US Holstein data have shown flat trends for heat tolerance in the first parity and declining trends for later parities, with an increased variance for the heat tolerance effect (Aguilar et al., 2010).

A broken line model using genomic information was implemented for the national dairy cattle evaluation in Australia (Nguyen et al., 2016). Practical comparisons of heat-tolerant and -intolerant animals in the United States using SNP effects derived in Australia showed only slight improvement in rectal temperature (0.1°C), with a 5% decline in production; the evaluation used only first-parity cow records (Jensen et al., 2022). In the United States, McWhorter et al. (2023) investigated responses of Holsteins to THI and found a threshold of 69 for milk production traits. Twenty years earlier, Ravagnolo and Misztal (2000a) reported a threshold of 72, indicating slightly increased sensitivity of cows to heat stress over the years. However, the environmental sensitivity may be even stronger assuming that heat abatement devices have improved, masking a substantial fraction of heat stress.

Aside from production traits, Ravagnolo et al. (2000b) showed a decline in fertility, measured as nonreturn rate after the first parity, when THI was over 72, with considerably reduced heritabilities. Analyzing nonreturn rates in 2 parities, Biffani et al. (2016) found twice as high genetic variance in the second than the first parity. However, results of fertility studies are affected by many factors that are not considered, including the voluntary waiting period, the presence of timed AI, and the use of heat abatement strategies in the farms. Another approach to analyze fertility under heat stress uses monthly variations on days open (Oseni et al., 2004). A genetic evaluation based on that concept showed that the most heat-tolerant bulls had 2 mo longer productive life and over 3% higher daughter pregnancy rate compared with the least heat-tolerant bulls (Pszczola et al., 2009), although their PTA for milk was 436 kg lower.

In addition to the broken line approach described above, random regression models based on functions such as Legendre orthogonal polynomials (Oliveira et al., 2019; Vinet et al., 2024) have also been used for modeling phenotypic variability of various traits across an environmental gradient without the need for defining a heat stress threshold. However, estimating parameters of complex models based on few records with extreme THI can lead to artifacts, especially that most changes in the parameters are expected after the threshold of heat stress while changes with a random regression model are over the entire THI range. Sánchez et al. (2009) implemented a very complex and compute-intensive model that estimated a different threshold for each herd. Ranking by models using fixed and variable thresholds was similar, indicating that the broken line model may be satisfactory for practical applications.

Although average daily THI is the most common environmental gradient used in heat tolerance studies, variability in daily THI or even other environmental variables (e.g., daily maximum temperature; dew point; thermal comfort index, overnight cooling extent) may be used, such as maximum THI per day or number of days with average temperature above a certain threshold. In addition to the environmental gradient, the critical period adopted for summarizing the environmental gradient values (e.g., average THI of 1 wk before each phenotypic measurement) needs to be evaluated based on the performance trait evaluated (e.g., milk production traits or fertility indicators). For example, an average of THI of 5 d before milk test was found best by Nguyen et al. (2016) for production traits. For fertility traits, Biffani et al. (2016) found that the best THI was an average of 4 d before the insemination for the first parity, and 3 d after the insemination for the second parity. Indices based on multiple day averages may have a small impact if the weather changes slowly.

Based on many studies, Misztal (2017) provided a hypothetical profile of a modern cow in response to increased THI (Figure 1). The profile includes 2 thresholds, like those postulated in a study on resource allocation (van der Waaij, 2004). First, the cow functions normally until the first THI threshold, which corresponds to the ability of the cow to dissipate heat associated with feed intake and metabolic heat production. Then, the production starts dropping slightly, with a rise of rectal temperature at a cost of lower fertility. Following the second threshold, the production decreases rapidly with increased risk of death or morbidity. The thresholds as well as actual changes are likely to be functions of the management. Intensive cooling will push both thresholds higher and rapid veterinary care would reduce mortality.

**Figure 1:**
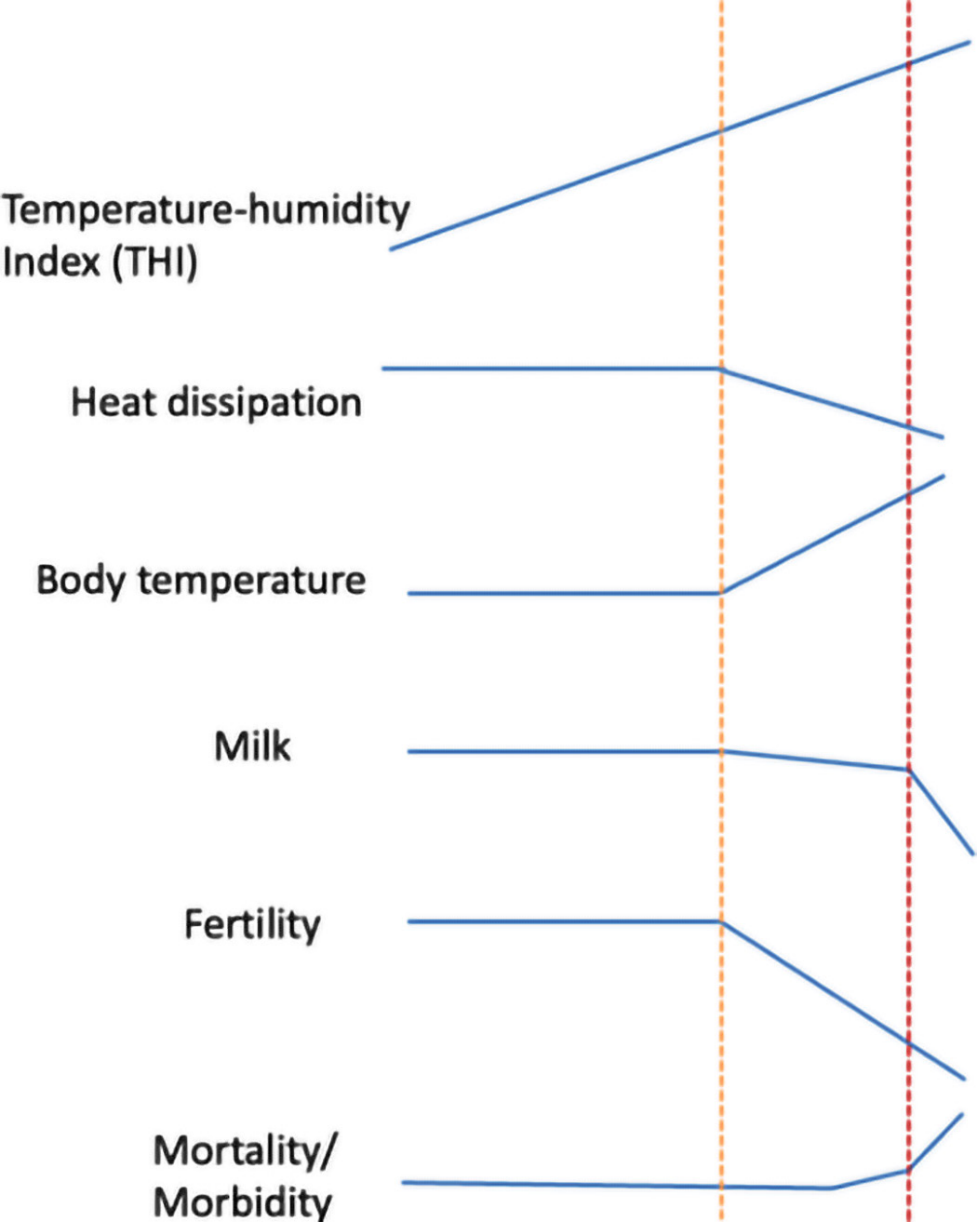
Profile of a high-producing modern cow under increasing THI. The orange and red dashed lines represent the first and second THI thresholds, respectively.

If PTA for heat tolerance is available, a desirable cow can be one of 3 types, as illustrated in Figure 2. Type 1) A high-production cow that is selected only on the regular PTA disregarding heat tolerance, likely showing low heat tolerance PTA. She would be a high producer under mild temperatures, with a decline under heat stress conditions. Under heat stress, she would be more likely to suffer from poor fertility, compromised health and welfare, and would be at high risk of death. Type 2) A heat-tolerant cow that is selected mainly on the heat tolerance PTA. Such a cow would have somewhat lower production than the high-producing cow, but would better maintain her production level under heat stress. Under a high level of heat stress, the cow would be more fertile and less prone to death than the high-production cow. Type 3) A hypothetical “resilient” cow that is high producing under mild temperatures, which at the onset of heat stress steeply reduces production to a level that allows her to maintain good fertility and reduce the risk of death, and would recover milk production rapidly after the heat stress is over. Which cow to choose depends on the length of the hot and humid season (long or short), the level of management (high or low), and the availability of veterinary care. In a survey in Spain (Martin-Collado et al., 2024) about the farmers' attitude to select for heat stress, heat tolerance was preferred, provided the production stayed high. Farmers in the United States tend to prefer high-production cows even if they require a high level of care (Tom Lawlor, US Holstein Association, Brattleboro, VT, personal communication).

**Figure 2:**
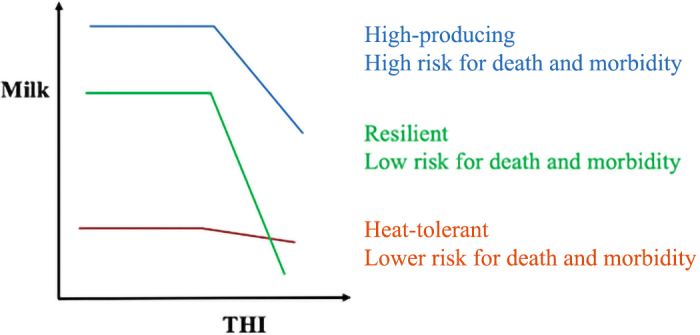
Milk production under increasing THI for a high-producing but high-risk cow, heat-tolerant but lower-producing cow, and hypothetical resilient cow who is high producing at lower THI but drastically lowers production at high THI.

When the hot season is long, high-producing herds must include effective heat mitigation devices. As they cannot tolerate low production during the mild season, they probably choose a high-producing cow, utilizing intensive management to maintain fertility, and veterinary care to minimize morbidity and mortality. Under low management, the heat-tolerant cow is attractive as she maintains a stable production with fewer or no heat mitigation devices, and better fertility and survival would reduce costs. The situation could be different in farms under a short period of heat stress. Even under high management, the heat mitigation devices are less justified, but a short period of heat stress could result in high costs. Such farms could benefit from a resilient cow (type 3).

Resilient animals have the capacity to be minimally affected by disturbances or to rapidly return to the state pertained before exposure to a disturbance (Berghof et al., 2019). With a disturbance caused by heat, resilience would mean a reduction in production during the heat stress to a comfortable level, and a resumption of production after the heat stress is over. A study modeled the ability of a cow to resume production after a short or long stress (Chen et al., 2023) and the results are unclear. Wang et al. (2022) looked at disturbances in dairy milk yield and found them to be an average of 19 d, after which the milk returned to normal. In general, the recovery is likely more complete with a shorter than longer period of heat stress. If this ability is under genetic control, it can be selected for.

In some studies, resilience was defined as consistency of phenotypes over a gradient, or a minimal residual variance (Poppe et al., 2022). A theory called canalization postulates that a function of the residual variance has a genetic component (Bodin et al., 2002). In a canalization model, each animal has 2 breeding values, one general and one for the residual dispersion. If the objective is to reduce environmental variability, the second breeding value should be considered. In a study in rabbits, lower residual variance was associated with higher production and better health (Garreau et al., 2008), which was understood as fewer disruptions due to diseases and other factors. In dairy cattle, Wang et al. (2022) looked at the log of the variance of test days relative to a standard lactation curve. The studied trait was found to be heritable with an estimate up to 0.25, and a larger variance was positively associated with many disorders. Chen et al. (2023) derived resilience traits in US Holstein based on daily milk yield residuals, including weighted occurrence frequency of yield perturbations, accumulated milk losses of yield perturbations, log-transformed variance, and lag-1 autocorrelation of daily milk yield residuals. All these traits are heritable and the genetic correlation results across lactations highlighted that different resilience indicators may capture additional biological mechanisms and should be considered as different traits in genetic evaluations. The authors also reported favorable genetic correlations of these resilience indicators with longevity and Net Merit Index. Guinan et al. (2024) showed profiles of “consistent” and “inconsistent” cows; production of consistent cows was very close to a standardized curve, whereas “inconsistent” cows showed many variations, mostly negative but some positive.

The effect of heat stress onset on consistency can vary depending on the model for analysis and the type of cow. Assume that consistency is a deviation from a standard lactation curve following cows in Figure 2. At the onset of heat stress, the heat-tolerant cow could show a small drop, a high-producing cow could show a bigger drop, and the resilient cow could show an even larger drop, but with a fast recovery. Assume that the deviations are from a model with the effect of herd-test-day, which includes the average effect of heat. Then, a heat-tolerant cow could show a positive deviation, a high-producing cow could show little deviation, and a resilient cow could show a negative deviation. Subsequently, the utility of consistency in identifying heat-tolerant or -resilient cows would be useful if fluctuations are classified as desirable (e.g., in response to heat stress to avoid problems) and undesirable (e.g., in response to disease). One example of resilience in beef cattle is the response of animals to heat stress with the removal of water access (Scharf et al., 2008). Cows stopped eating, reducing heat generation and fluid loss, and recovered a few days after returning to normal.

Studies indicated that most heat-tolerant animals identified by the broken line model are superior for secondary traits, including fertility and survival (Bohmanova et al., 2008; Nguyen et al., 2016; Jensen et al., 2022), indicating positive correlations between those traits. Direct correlations between heat tolerance and fitness and conformation traits are not available. An informal characteristic of a heat-tolerant animal would be overall high production, high fertility, and low morbidity and mortality. If direct evaluations for heat tolerance are not available, one option is multitrait selection, as practiced now, with increased weight on fertility, health, livability, and possibly other indicator traits. The weights would be farm-dependent, probably with larger weights for secondary traits in farms with less intensive management.

As global warming is likely to intensify, there is a question of whether the selection described previously will be sufficient to ensure animal welfare. Improvements in the efficiency of animal production are possibly due to a joint improvement in management and genetics, although at a cost of resilience (Misztal and Lourenco, 2024). Improved management compensates for deterioration in secondary traits caused by mainly selection on higher heritability traits. Therefore, with increased temperatures, the management of heat stress will have to keep improving, possibly following the pace of global warming. In locations where the management is difficult or too expensive, the dairy production may become unprofitable, for example, as has already occurred in the states of Alabama, Mississippi, and Louisiana.

The traits of heat tolerance may be different in farms with low and high levels of heat stress and, therefore, with likely different heat abatement management. If so, a case can be made for separate evaluations in these environments. Bohmanova et al. (2008) ran a genetic evaluation for 2 regions separately: southeast with intensive heat management and northwest with less intensive or no heat management. The correlations of HTBV for high reliability sires were 0.8, indicating little reranking in the 2 regions. Subsequently, the effect of heat tolerance seems to be similar across environments, although its magnitude can be different. A similar phenomenon was observed for fertility across the United States (Paul VanRaden, Animal Genomics and Improvement Laboratory-USDA, Beltsville, MD, personal communication), where despite large differences in environments and management of fertility across the United States, the trait was similar, allowing for a single national evaluation.

Overall, selecting for heat tolerance and resilience may be possible; however, identifying proper phenotypes to accurately deem a cow resilient to environmental disturbances is still challenging. Because of the multiple biological processes involved in heat stress response (Dikmen et al., 2012; Luo et al., 2022), effective selection for improved heat tolerance could consider indicator traits related to both heat gain and heat dissipation processes. Further improvement is expected in the near future when precision phenotyping technologies become widespread, and breeding companies realize the economic value of heat tolerance on the overall sustainability of the dairy industry.
